# Gas Chromatography Coupled to High Resolution Time-of-Flight Mass Spectrometry as a High-Throughput Tool for Characterizing Geochemical Biomarkers in Sediments

**DOI:** 10.1155/2018/2560498

**Published:** 2018-12-02

**Authors:** Hector Henrique Ferreira Koolen, Clécio Fernando Klitzke, Joe Binkley, Jeffrey Patrick, Ana Cecília Rizatti de Albergaria-Barbosa, Rolf Roland Weber, Márcia Caruso Bícego, Marcos Nogueira Eberlin, Giovana Anceski Bataglion

**Affiliations:** ^1^ThoMSon Mass Spectrometry Laboratory, Institute of Chemistry, University of Campinas (UNICAMP), 13083-970, Campinas, SP, Brazil; ^2^Metabolomics and Mass Spectrometry Research Group, Amazonas State University (UEA), 69065-001, Manaus, AM, Brazil; ^3^LECO Corporation, 49085, St. Joseph, MI, USA; ^4^Laboratory of Marine Geochemistry, Geoscience Institute, Federal University of Bahia (UFBA), 40170-020, Salvador, BA, Brazil; ^5^Marine Organic Chemistry Laboratory, Oceanographic Institute, University of São Paulo (USP), 05508-120, São Paulo, SP, Brazil; ^6^Department of Chemistry, Federal University of Amazonas (UFAM), 69077-000, Manaus, AM, Brazil

## Abstract

The performance of gas chromatography coupled to high-resolution time-of-flight mass spectrometry (GC-HRTofMS) for characterizing geochemical biomarkers from sediment samples was evaluated. Two approaches to obtain the geochemical biomarkers were tested: (1) extraction with organic solvent and subsequent derivatization and (2) in-situ derivatization thermal desorption. Results demonstrated that both approaches can be conveniently applied for simultaneous characterization of many geochemical biomarkers (alkanes, alkanols, sterols, and fatty acids), avoiding conventional time-consuming purification procedures. GC-HRTofMS reduces both sample preparation time and the number of chromatographic runs compared to traditional methodologies used in organic geochemistry. Particularly, the approach based on in-situ derivatization thermal desorption represents a very simple method that can be performed in-line employing few milligrams of sediment, eliminating the need for any sample preparation and solvent use. The high resolving power (*m*/Δ*m*_50%_ 25,000) and high mass accuracy (error ≤ 1 ppm) offered by the “zig-zag” time-of-flight analyzer were indispensable to resolve the complexity of the total ion chromatograms, representing a high-throughput tool. Extracted ion chromatograms using exact* m/z* were useful to eliminate many isobaric interferences and to increase significantly the signal to noise ratio. Characteristic fragment ions allowed the identification of homologous series, such as alkanes, alkanols, fatty acids, and sterols. Polycyclic aromatic hydrocarbons were also identified in the samples by their molecular ions. The characterization of geochemical biomarkers along a sedimentary core collected in the area of Valo Grande Channel (Cananéia-Iguape Estuarine-Lagunar System (São Paulo, Brazil)) provided evidences of environmental changes. Sediments deposited before opening of channel showed dominance of biomarkers from mangrove vegetation, whereas sediments of the pos-opening period showed an increase of biomarkers from aquatic macrophyte (an invasive vegetation).

## 1. Introduction

Organic matter (OM) is ubiquitously present in all natural waters and sediments, playing a central role in many environmental processes such as global carbon and nutrient cycles [1]. It can encompass many classes of compounds, such as alkanes, alkanols, sterols, and fatty acids, which are widely considered as geochemical biomarkers [2–4].

The organic geochemistry field comprehends diversified studies about OM in sediments; thus the biomarkers of interest may vary depending on the focus. Compounds classes, such as* n*-alkanes, alkanols, sterols, and fatty acids, are valuable to identify changes in the relative contributions of autochthonous and allochthonous OM sources to the sedimentary record over historical time-scale [5, 6]. They have also been used to assess changes in biogeochemical processes related to aquatic productivity [7], diagenetic alterations [8], and climate changes [9]. Hydrocarbons, such as* n*-alkanes, hopanes, and steranes, are also important for investigating contamination by crude oil and its derivatives in recent sediments [10, 11]. Sterols are of special interest to evaluate the contamination levels from domestic sewage discharge [12, 13]. Additionally, several studies have employed a comprehensive approach based on the assessing of diverse classes of geochemical biomarkers to increase the reliability of the interpretations [14, 15].

A classic methodology based on the traditional gas chromatography coupled to mass spectrometry (GC-MS) technique is widely employed for characterizing geochemical biomarkers in sediment samples. Briefly, an extraction step is followed by purification of the raw organic extract on silica/alumina columns to yield fractions with fewer compounds [11, 14, 15]. The need of a purification step is due to the chemical complexity of organic matter that would complicate the separation from raw extract by the traditional GC-MS technique, resulting in unresolved complex mixture (UCM) [16]. This term refers to a raised “baseline hump” in chromatograms composed by a mixture of compounds unresolved by capillary columns [17]. The problem related to UCM regions can be partly resolved by the previous purification step, although this procedure requires large solvent quantity and is time-consuming.

High-resolution mass spectrometry (HRMS) coupled to GC is rarely used in environmental analysis, even though its benefits are well known [18, 19]. For instance, the advantages of GC-HRMS for qualitative and quantitative environmental analyses have been demonstrated for persistent organic compounds in sediments [18, 20]. Such studies have reported the elimination of matrix problems using accurate mass measurements [21, 22]. In this sense, gas chromatography coupled to high-resolution time-of-flight mass spectrometry (GC-HRTofMS) seems attractive to simultaneously characterize diverse classes of geochemical biomarkers from the raw extract without any time-consuming purification step on alumina/silica columns. There is also a possibility of chemical characterization directly from sediment samples using thermal desorption interfaced to GC-HRTofMS. That technique is based on the thermal desorption of compounds from a small amount (mg) of sediment, and then they are transferred to the GC inlet by a carrier gas [23, 24]. A similar procedure is based on pyrolysis, in which occurs thermal degradation of macromolecular OM, such as biopolymer, generating low-molecular weight products [25]. Thermal desorption, however, allows the characterization of volatile and semivolatile compounds in their intact form using lower temperature than those commonly used in pyrolysis [23, 24]. Despite the advantages of both GC-HRTofMS and thermal desorption, these approaches are rarely used together in organic geochemistry of recent sediments.

The main objective of the present study was to investigate the performance of GC-HRTofMS in characterizing simultaneously diverse classes of geochemical biomarkers. For that, we tested and compared two approaches to obtain geochemical biomarkers: (1) extraction with organic solvent and subsequent derivatization and (2) in-situ derivatization thermal desorption. Then, as a field-testing application, geochemical biomarkers were characterized along a sedimentary core from the Cananéia-Iguape estuarine-lagoonal system (Brazil) to investigate changes in OM sources through the depositional period. The study area is in the World Heritage List under natural criteria of the United Nations Educational Scientific and Cultural Organization (UNESCO) because of its extensive areas of mangrove and Atlantic forest. However, an opening of an artificial channel (Valo Grande Channel) may had disturbed organic matter cycle.

## 2. Experimental

### 2.1. Study Area and Samples

A sedimentary core (198 cm) was collected in the Cananéia-Iguape Estuarine-Lagunar System (São Paulo, Brazil), more specifically in front of the Valo Grande Channel area, at latitude and longitude of 24°45′2.45′′ S and 47°37′3.70′′ W, respectively, with the aid of a Rossfelder VT-1 vibracorer. The study area and sampling station are shown in Figure S1 in Supplementary Material. The sedimentary core was sampled continuously at intervals of 2.0 cm immediately after collection. The samples were frozen at -18°C and freeze-dried at -50°C and pressure of 270 mbar with a Thermo Savant Modulyo D freeze dryer (Thermo Electron Corporation, Madison, USA). That environmental system is located in the southeastern Brazilian coast and consists of a complex of lagoonal channels with a broad and relatively well-preserved Atlantic forest and mangrove vegetation. The Cananéia-Iguape Estuarine-Lagunar System is considered by the UNESCO as a Biosphere Reserve. However, environmental changes have occurred in that system in the last two centuries because of the artificial opening of a channel (Valo Grande Channel) connecting the Ribeira de Iguape River to the lagoonal system. The original dimensions (4.4 m wide and 2.0 m deep) of the channel rapidly increased and currently are about 250 m of wide and 7 m of deep [26]. The average percentage of clay, silt, and sand of the sediment samples were 5.4, 43.4, and 51.1, respectively, whereas average percentage of total organic carbon (TOC) was 1.92%. The content of fine particles (silt and clay) as well as TOC presented higher values in the first 138 cm of the sedimentary core.

### 2.2. Extraction with Organic Solvent and Subsequent Derivatization

The freeze-dried sediment samples were macerated, and 20 g was extracted using the microwave system MarsX (CEM Corporation, Mathews, USA) operating at 1600 W. The extractions were performed with 50 mL of* n*-hexane and dichloromethane (1:1, v/v) with pressure and temperature gradients reaching 200 psi and 85°C in 5 min. The system was kept isothermal for 15 min, returning to the initial pressure and temperature conditions. The organic extracts were evaporated to 1.0 mL, and then an aliquot of 50.0 *μ*L was derivatized using bis(trimethylsilyl) trifluoroacetamide (BSTFA) with 1% trimethylchlorosilane (TMCS) for 120 min at 70°C. After reaction, the residual reagents were evaporated to dryness with a gentle flow of nitrogen stream and the extract was redissolved to exactly 200 *μ*L of* n*-hexane. All solvents used were of HPLC grade, purchased from Mallinckrodt Chemicals, and used as received.

### 2.3. In-Situ Derivatization Thermal Desorption

An amount of 20 mg of the homogenized and freeze-dried sediment sample was added into a small quartz tube, which is heated up with a pyroprobe using a platinum filament coil. Quartz wool was properly packed at the top and bottom to avoid possible leakage, or the sample being blown out. An aliquot of 10 *μ*L of a 25% methanolic tetramethylammonium hydroxide (TMAH) solution was added to the sample inside the quartz tube to provide deeper insights in alkanol, sterol, and fatty acid compositions. Using TMAH, hydroxyl groups of carboxylic acids and alcohols were in-situ derivatized in the same step of thermal desorption and converted to the corresponding methyl esters and methyl ethers, respectively. The compounds present in the sediment samples were thermally desorbed at 400°C during 20 s, using a CDS Analytical Pyroprobe® 5200 (Chemical Data Systems, Oxford, USA) coupled to a Leco Pegasus® GC-HRT MS. Pyroprobe interface, valve oven and transfer line were at 300°C. Helium was used as the carrier gas (1.0 mL min^−1^) to transfer the analytes during the course of thermal desorption directly to the GC inlet during 4 min at 300°C.

### 2.4. GC-HRTofMS Analysis

The equipment used, Leco Pegasus® GC-HRT MS, consists of an Agilent 7890 GC (Agilent Technologies, Palo Alto, USA) and a “zig-zag” TOF analyzer (LECO Corporation, St. Joseph, USA). A Restek Rxi-5MS column (30 m, 0.25 mm I.D., 0.25 *μ*m film thickness) was used at initial temperature of 50°C for 2 min, raised to 320°C with the rate 6°C min^−1^, and maintained for 20 min. A split ratio of 50:1 was used to inject liquid samples and splitless mode was used for the thermal desorption experiments. Helium was used as the carrier gas with constant flow rate of 1.0 mL min^−1^. GC inlet and transfer line to the ion source were at 300 and 320°C, respectively. Electron ionization (EI) source was at 70 eV, 3 mA, and 250°C. Data were recorded in full scan mode from 50-550* m/z* with resolving power (*m*/Δ*m*_50%_) > 25,000 and mass error < 1.0 ppm using acquisition rate of 6 spectra s^−1^ and extraction frequency of 1.75 kHz. Data were acquired and processed using Leco ChromaTOF HRT software (LECO Corporation, St. Joseph, USA) and chromatographic peaks were identified based on spectra from NIST library, retention time of standards, and elution order.

## 3. Results and Discussion

**Figure 1:**
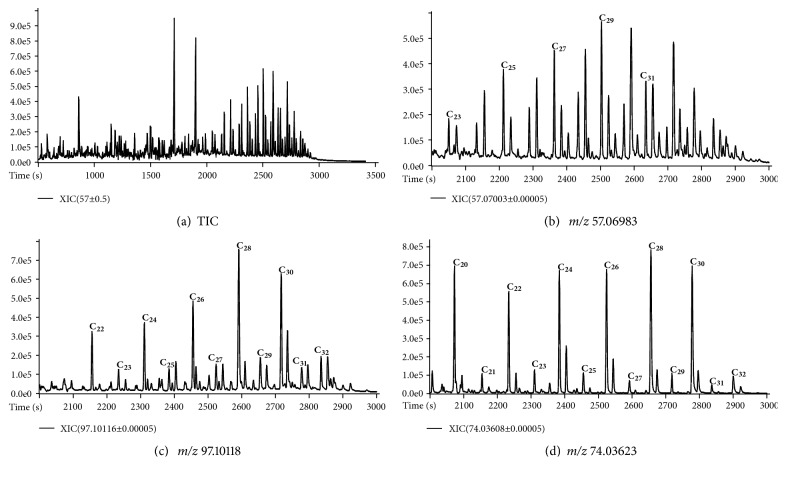
(a) TIC obtained by using the approach based on in-situ derivatization thermal desorption for a sediment sample; (b) expansion (2000-3000 s) of XIC for* m/z* 57.06983 relative to the series of* n*-alkanes, (c) expansion (2000-3000 s) of XIC for* m/z* 97.10118 relative to the series* n*-alkanols, and (d) expansion (2000-3000 s) of XIC for* m/z* 74.03623 relative to the series of* n*-fatty acids.* n* in C*n* is the C number.

A representative total ion chromatogram (TIC) obtained by using the approach based on in-situ derivatization thermal desorption is shown in Figure 1.

Note the difficulty in resolving the entire wide range of compounds present in sediment samples. Although the complexity of the chromatogram, chemical characterization of important geochemical biomarkers can be achieved because of the high resolution offered by the “zig-zag” TOF analyzer. The extracted ion chromatograms (XIC) for characteristic exact* m/z* and the elution patterns allowed the identification of aliphatic compounds classes, such as* n*-alkanes,* n*-alkanols, and* n*-fatty acids, as shown in Figure 1.

The class of* n*-alkanes was identified by extracting chromatograms for the* m/z* 57.06983 (exact mass: 57.06988, error: -0.87 ppm), which corresponds to the common fragment CH_3_CH_2_CH_2_CH_2_^+^. Note that a series of* n*-alkanes obtained via thermal desorption approach is not present as doublets of* n*-alk-1-ene/*n*-alkane. Such doublets are formed through the homolytic cleavage of long alkyl chains of aliphatic biopolymers upon pyrolysis [27]. The absence of* n*-alk-1-ene/*n*-alkane doublets shows that thermal desorption is able to characterize the aliphatic composition of sedimentary OM without degrading biopolymers, which would complicate interpretation of results. The TMAH reagent converts fatty acids and alkanols in their methyl ester and methyl ether derivatives, respectively, which are more easily analyzed by GC. The classes of* n*-alkanols and* n*-fatty acids were identified by extracting chromatograms for the* m/z* 97.10110 (exact mass: 97.10118, error: -0.82 ppm) and* m/z* 74.03617 (exact mass: 74.03623, error: -0.81 ppm), respectively. The ion at* m/z* 97.10118 is due to the fragment CH_3_(CH_2_)_4_CHCH^+^, which allows extracting only* n*-alkanols without interference of* n*-fatty acids that are coeluted. The ion at* m/z* 74.03623 is due to the fragment CH_3_OCOHCH_2_^+^ that was generated by McLafferty rearrangement ion for methylated fatty acids.

**Figure 2:**
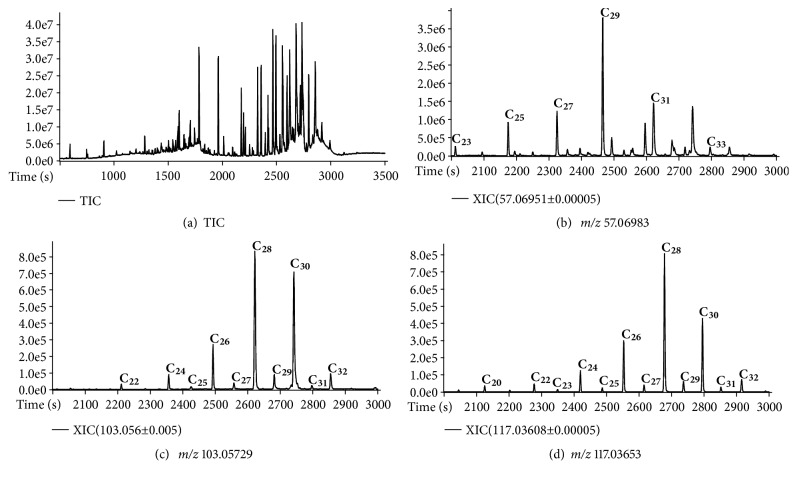
(a) TIC obtained by using the approach based on extraction with organic solvent and subsequent derivatization for a sediment sample; (b) expansion (2000-3000 s) of XIC for* m/z* 57.06983 relative to the series of* n*-alkanes, (c) expansion (2000-3000 s) of XIC for* m/z* 103.05729 relative to the series* n*-alkanols, and (d) expansion (2000-3000 s) of XIC for* m/z* 117.03653 relative to the series of* n*-fatty acids.* n* in C*n* is the C number.

For the approach using extraction with organic solvent and subsequent derivatization, a representative TIC is shown in Figure 2. The class of* n*-alkanes was also identified by extracting chromatograms for the* m/z* 57.06983 (exact mass: 57.06988, error: -0.87 ppm). Considering that the conventional reagent BSTFA was used for derivatization, the classes of* n*-alkanols and* n*-fatty acids were identified by extracting chromatograms for the* m/z* 103.05729 (exact mass: 103.05737, error: -0.77 ppm) and* m/z* 117.03663 (exact mass: 117.03653, error: -0.85 ppm), respectively. These ions correspond to the fragments (CH_3_)_3_SiOCH_2_^+^ and (CH_3_)_3_SiOCO^+^ generated by *α*-cleavage from the molecular ions of* n*-alkanols and* n*-fatty acids, respectively.

Usually, chromatograms for* n*-alkanes,* n*-alkanols, and* n*-fatty acids classes are obtained by individual chromatographic runs of purified fractions from raw extract. In this study, the results obtained for both approaches show that GC-HRTofMS allows characterizing simultaneously* n*-alkanes,* n*-alkanols, and* n*-fatty acids, reducing both sample preparation time and number of chromatographic runs, which is a novel aspect in the organic geochemistry field. Particularly, the approach based on in-situ derivatization thermal desorption allows characterizing directly the free lipids fraction from milligrams of sediment, requiring no organic solvents and leading to elimination of all issues related to their use and disposal, which is also a novel aspect in the organic geochemistry field. However, this approach may not be adequate for the analysis of sediments with very low content of organic carbon; thus the approach based on extraction with organic solvent and subsequent derivatization may be an option in these cases. We highlight that the high resolving power (*m*/Δ*m*_50%_ 25,000) and high mass accuracy (error ≤1 ppm) offered by the “zig-zag” time-of-flight analyzer were indispensable to resolve the complexity of TIC. By using GC-HRTofMS, many isobaric interferences were eliminated and the signal to noise ratios (S/N) were significantly increased in the XIC from exact* m/z* in comparison to those from unitary* m/z* for all geochemical biomarker classes identified in both approaches. It is also noteworthy to mention that, for a same sediment sample, results found by in-situ derivatization thermal desorption are equivalent to those obtained by extraction with organic solvent and subsequent derivatization. Therefore, thermal degradation used in this study can desorb free lipids while compounds bonded to insoluble polymeric material remain preserved in the sediment.

**Figure 3:**
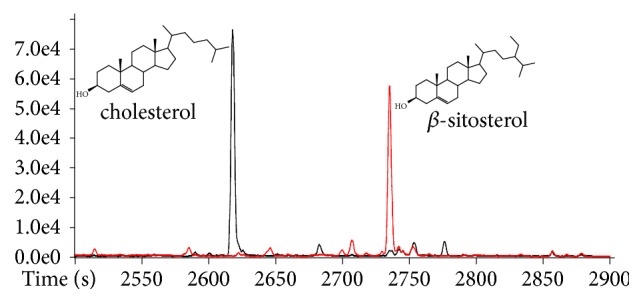
Expansion (2500-2900 s) of XIC for* m/z* 368.34354 and* m/z* 396.37481, which corresponds to cholesterol (molecular mass: 386.35432 u) and *β*-sitosterol (molecular mass: 414.38562 u), respectively. Mass spectra of cholesterol and *β*-sitosterol are shown in Figure S2 in Supplementary Material.

Sterols are also geochemical biomarkers commonly used in the assessment of OM sources in aquatic environments and can be detected by extracting chromatograms for some key fragment ions, as shown for cholesterol and *β*-sitosterol in Figure 3.

For the approach based on in-situ derivatization thermal desorption, sterols are identified by extracting fragment ions correspondent to water loss from their intact molecules or methanol from derivatized ones, as shown in Figure 3 for cholesterol (exact mass: 368.34375, error: -0.57 ppm) and for *β*-sitosterol (exact mass: 396.37505, error: -0.60 ppm). For the approach based on the extraction with organic solvent and subsequent derivatization using the conventional reagent BSTFA, those ions correspond to the fragments formed after the loss of the group (CH_3_)_3_SiOH. Beyond cholesterol and *β*-sitosterol, cholestanol, brassicasterol, campesterol, stigmasterol, and stigmastanol were also identified based on exact* m/z* of such fragment ions and elution order. Triterpenols, such as lupeol, *α*-amyrin, and *β*-amyrin, presented fragmentation behavior similar to sterols.

**Figure 4:**
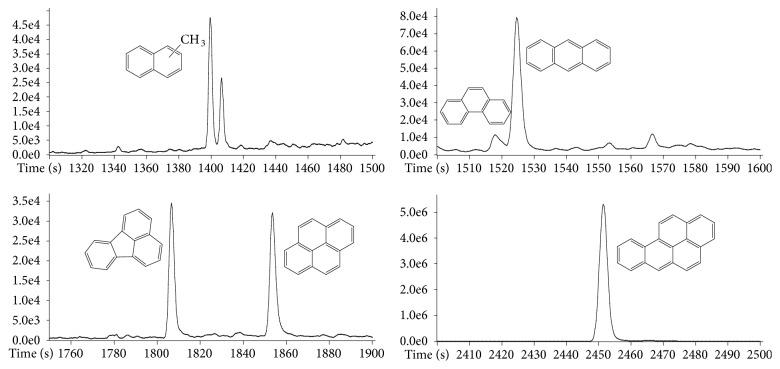
Expansion of XIC for* m/z* 142.07760 (methyl naphthalene),* m/z* 178.07759 (phenanthrene/anthracene),* m/z* 202.07757 (fluoranthene/pyrene), and* m/z* 252.09335 (benzo[a]pyrene).

**Figure 5:**
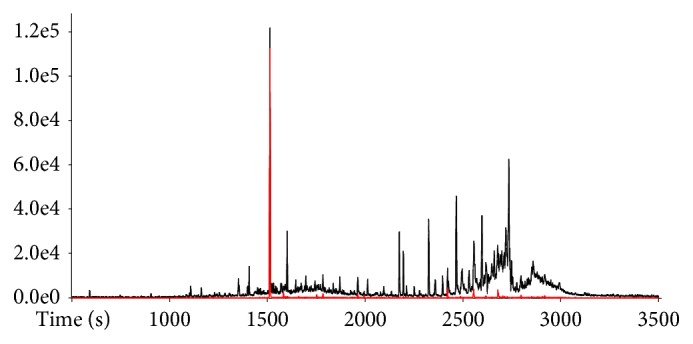
XIC for C_2_ naphthalene in low resolution (*m/z* 156, dark chromatogram) and high resolution (*m/z* 156.0559, red chromatogram).

Polycyclic aromatic hydrocarbons (PAH) are another class of compounds that may be present in sediments, especially those from industrial areas. They are emitted mainly during incomplete burning of coal, oil, gas, coke, wood, garbage, or other organic materials. These sources are called pyrogenic, whereas crude oil, refined petroleum, coal, coal tar, pitch, and asphalts represent petrogenic sources. Natural sources, including volcanism and diagenetic processes, are also possible [3, 28, 29]. Several PAH can be identified by extracting chromatograms for their molecular ions, such as* m/z* 142.07760 (methyl naphthalene, exact mass: 142.07770, error: -0.70 ppm),* m/z* 178.07759 (phenanthrene/anthracene, exact mass: 178.07770, error: -0.62 ppm),* m/z* 202.07757 (fluoranthene/pyrene, exact mass: 202.07770, error: -0.64 ppm), and* m/z* 252.09311 (benzo[a]pyrene, mass exact: 252.09335, error: -0.95 ppm), as shown in Figure 4. Also, the effectiveness of high resolving power and mass accuracy offered by the “zig-zag” time-of-flight analyzer for PAH is shown in Figure 5 with the comparison between ion chromatograms extracted in high and low resolution as measured by typical quadrupole analyzer.

Figure 5 shows that the high resolving power and mass accuracy were also important to eliminate many isobaric interferences and increase S/N for PAH.

**Figure 6:**
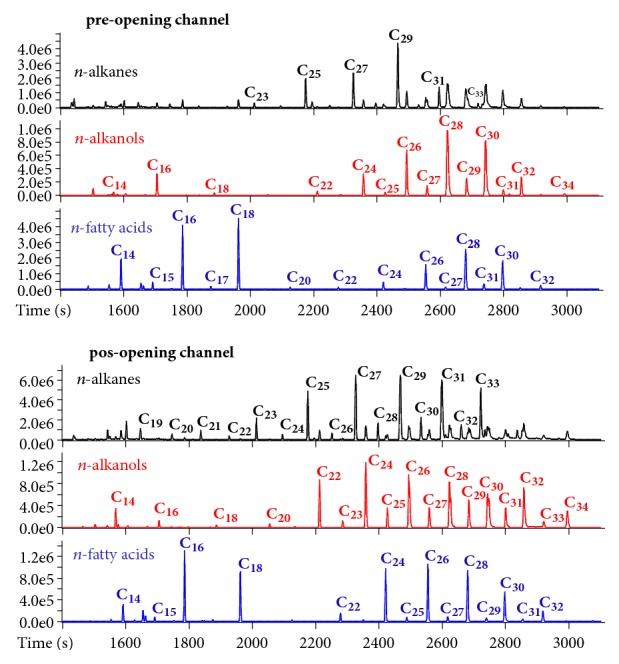
Expansion (1500-3000 s) of XIC for* n*-alkanes,* n*-alkanols, and* n*-fatty acids for representative samples of both pre-opening and pos-opening periods of the Valo Grande Channel in the Cananéia-Iguape Estuarine-Lagunar System.* n* in C*n* is the carbon number.* n*-alkanes,* n*-alkanols, and* n*-fatty acids series were obtained by extracting chromatograms for the* m/z* 57.06983, 103.05729, and 117.03663, respectively.

As a proof of concept of the ability of GC-HRTofMS as a high-throughput tool in organic geochemistry, OM characterization was done along a sedimentary core to assess the historical record in the area of the Valo Grande Channel, artificially created in the Cananéia-Iguape Estuarine-Lagunar System in 1852 [26].* n*-Alkanes,* n*-alkanols, and* n*-fatty acids are straight chain hydrocarbons produced by many organisms and the dominant chain lengths and carbon number distributions vary depending on the source organism [25]. The long-chain* n*-alkanes (C_27_ to C_31_) and* n*-alkanols and* n*-fatty acids (C_26_ to C_32_) are the main components of epicuticular waxes of higher plants, whereas algae are dominated by shorter-chain* n*-alkanes (C_17_ to C_21_) and* n*-alkanols and* n*-fatty acids (C_14_ to C_18_). The mid-chain* n*-alkanes (C_21_ to C_25_) and* n*-alkanols and* n*-fatty acids (C_20_ to C_24_) are dominant in aquatic plants, such as macrophytes [30, 31]. Profiles of* n*-alkanes,* n*-alkanols, and* n*-fatty acids for samples from the sedimentary core were obtained by extracting chromatograms for the* m/z* 57.06983, 103.05729, and 117.03663, respectively, as shown is Figure 6.

Figure 6 shows that changes in the sources of OM delivered to the sedimentary core of the Valo Grande Channel can be detected by the distribution of* n*-alkanes,* n*-alkanols, and* n*-fatty acids.* n*-Alkanes series for recent sediments usually exhibits strong odd over even carbon number predominance, whereas* n*-alkanols and* n*-fatty acids exhibit strong even over odd carbon number predominance [30]. The* n*-alkanes series in the sedimentary core showed unimodal distribution ranging from C_19_ to C_33_, with strong odd over even carbon number predominance (Figure 6). The* n*-alkanols and* n*-fatty acids series ranged from C_14_ to C_34_ and C_14_ to C_32_, respectively, with strong even over odd carbon number predominance (Figure 6). Such distribution profiles are typical of areas with predominance of natural sources of organic matter [30], indicating that anthropogenic sources are negligible in the studied area.

The* n*-alkanes series in the sediments correspondent to the pre-opening channel period showed predominance of C_29_ (Figure 6), with similar distribution to that observed for the species Laguncularia racemosa (white mangle) and Rhizophora mangle (red mangle) [32]. In fact, these mangle species are the most abundant in the Cananéia-Iguape Estuarine-Lagunar System that present extensive areas of mangrove vegetation [13]. The* n*-alkanes distribution observed for the sediments correspondent to the pos-opening period, however, presented higher relative contribution of the mid-chain* n*-alkanes, C_21_, C_23_, and C_25_, and also similar contribution of C_27_, C_29_, and C_31_ (Figure 6). The* n*-alkanes distribution observed for sediments of the pos-opening period was similar to that observed for leaves of the macrophyte Spartina alterniflora and for sediments subject to that vegetation [32]. Other submerged and floating macrophytes also present profiles of* n*-alkanes with similar contribution of the homologous from C_23_ to C_29_ [31, 33, 34]. An increase of aquatic macrophyte has been observed in the Cananéia-Iguape Estuarine-Lagunar System lately and associated with the opening of the Valo Grande Channel [13].

The* n*-alkanols and* n*-fatty acids series also showed changes in their distribution profiles between the two deposition periods. The* n*-alkanols and* n*-fatty acids series in the sediments correspondent to the pre-opening channel period showed predominance of C_26_, C_28_ and C_30_ with a Gaussian-like distribution (Figure 6). Similar profile for* n*-alkanols was observed for sediments from the Pichavaram mangrove complex, located between two estuaries in India [35]. Similar profiles of both* n*-alkanols and* n*-fatty acids were also observed for sediments from estuaries where the mangrove vegetation,* Rhizophora mangle* (red mangle) and* Avicennia germinans* (black mangle), is dominant [36]. In contrast, in the sediments correspondent to the pos-opening channel period, the profiles of* n*-alkanols and* n*-fatty acids showed higher relative contribution of the mid-chain homologous, C_22_, C_24_, and C_26_. Profiles of distribution of both* n*-alkanols and* n*-fatty acids presenting high contribution of these mid-chain homologous have been observed for a variety of macrophytes aquatic [31].

**Figure 7:**
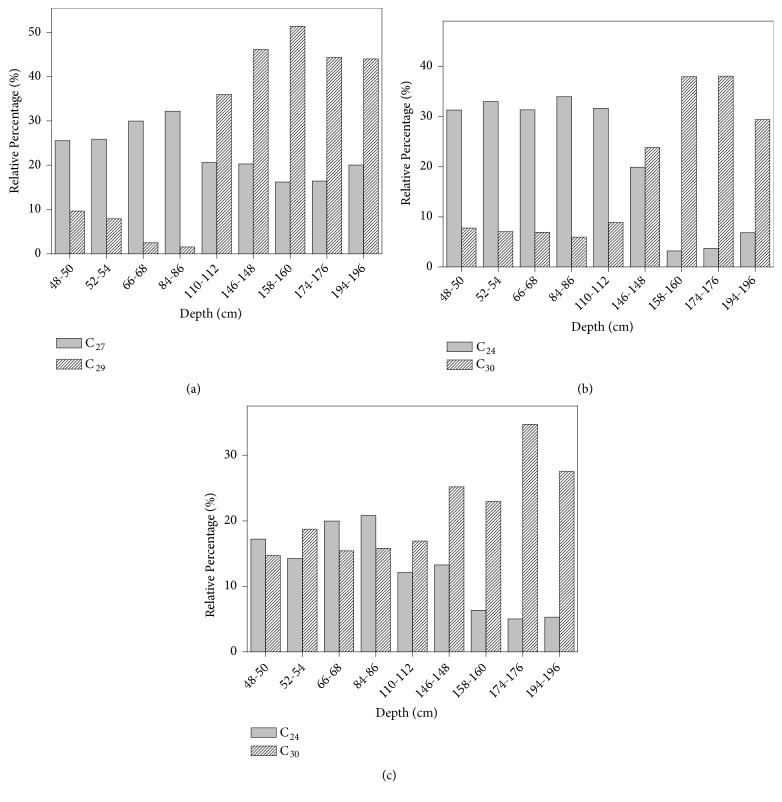
Variations of the relative abundances of* n*-alkanes C_27_ and C_29_ (a),* n*-alkanols C_24_ and C_30_ (b), and* n*-fatty acids C_24_ and C_30_ (c), along the sedimentary core collected at Valo Grande Channel in Cananéia-Iguape Estuarine-Lagunar System. Correspondence of sediment depth and year: 48-50 cm (1976), 52-54 cm (1972), 66-68 cm (1956), 84-86 cm (1925), 110-112 cm (1887), 146-148 cm (1816), 158-160 cm (1796), 174-176 cm (1776), 194-196 cm (1725).

The geochemical biomarker variations as function of depth can be better visualized by choosing a homologous of each class to represent terrestrial vegetation input and another to represent aquatic vegetation input, as shown in Figure 7.

Results presented in Figures 6 and 7 indicate that the reduction of the long-chain homologous dominant in higher plants is accompanied by the increase of the mid-chain ones, which are dominant in floating and submerged aquatic plants. Thus, the results reveal that the sediments deposited in the pre-opening channel period are characterized by the strong predominance of biomarkers of higher plants (mangrove vegetation), whereas the sediments deposited in the pos-opening channel period present an increase of biomarkers of aquatic macrophytes. In addition, Figure 7 indicates that the more abrupt changes in the relative abundance of biomarkers of different types of vegetation occur around 110-112 cm.

**Figure 8:**
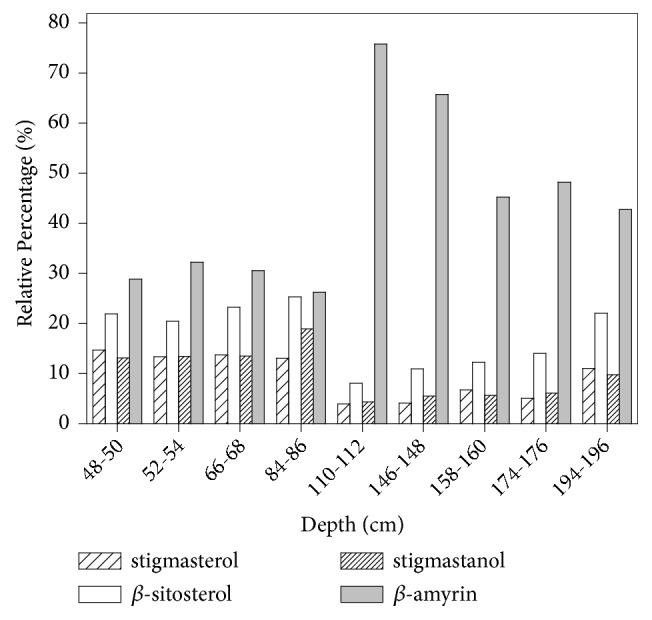
Variations in the relative abundances of some sterols and *β*-amyrin along the sedimentary core collected at Valo Grande Channel in Cananéia-Iguape Estuarine-Lagunar System. Correspondence of sediment depth and year: 48-50 cm (1976), 52-54 cm (1972), 66-68 cm (1956), 84-86 cm (1925), 110-112 cm (1887), 146-148 cm (1816), 158-160 cm (1796), 174-176 cm (1776), 194-196 cm (1725).

A previous study concerned to anthropogenic metal input in this estuarine-lagoonal system observed an abrupt increase of the concentration of Cr, Cu, Zn, and Sc in the same depth [26]. The authors also determined the sedimentation rates via ^210^Pb gamma spectrometry. Based on this analysis, the sedimentary core covers the period between 1723±15 and 2008. The sediment portion 110-112 cm, which corresponds to approximately 35±8 years after the conclusion of construction of the Valo Grande Channel (1827-1852), readily evidences the increase of biomarkers from aquatic vegetation. The channel construction resulted in an increase of freshwater flow from the Ribeira de Iguape River to the estuarine-lagoonal system, creating proper conditions for the reproduction of aquatic macrophytes [35]. Thus, the first period represents an environment subject to OM input from terrestrial plants, Atlantic Forest and mangrove vegetation. In a second period, a similar contribution of OM from terrestrial and aquatic vegetation is due to the invasive species of macrophytes. Equivalent discussion can be raised by the variations in the relative abundances of some sterols and *β*-amyrin as function of depth along the sedimentary core collected in Valo Grande Channel, as shown in Figure 8.

The compounds stigmasterol, *β*-sitosterol, and stigmastanol showed higher relative contribution in the pos-opening channel period, whereas the triterpenol *β*-amyrin was strongly dominant in the pre-opening channel period. Phytosterols, such as stigmasterol and *β*-sitosterol, are produced by plants in general, including aquatic macrophytes [14]. The triterpenol *β*-amyrin, however, is considered a highly specific biomarker for higher plants and its abundant presence in sediments confirms the widespread input of terrestrial OM [4, 14]. High abundance of *β*-amyrin was observed for sediments from estuaries dominated by mangrove vegetation [36]. The change in the relative contribution of such sterols and *β*-amyrin corroborates those observed for the classes of* n*-alkanes,* n*-alkanols, and* n*-fatty acids, indicating that MO input from aquatic vegetation has increased significantly after opening of the Valo Grande Channel.

Alkylated homologous PAH, such as methylnaphthalene (C_1_), dimethylnaphthalene (C_2_), and trimethylnaphthalene (C_3_), phenanthrene, anthracene, fluoranthene, pyrene, and benzo[a]pyrene were detected in all samples along the sedimentary core. These compounds have been classified as priority pollutants by the United States Environmental Protection Agency (USEPA) because of their toxic, carcinogenic, and mutagenic characteristics [30]. Source appointment of PAH in aquatic environments is difficult because of the multiples possible sources. Petrogenic PAH are predominantly of low molecular weight and alkylated, whereas pyrolitic HPA are dominated by the high molecular weight compounds [37]. The sediment samples deposited in the pre-opening channel period presented a mixture of low and high molecular weight and alkylated PAH with similar proportion, whereas those deposited in the pos-opening period presented an abrupt increase of the contribution of benzo[a]pyrene that is a five-member PAH well-known by the International Agency for Research on Cancer (IARC) as carcinogenic to human beings (group 1) [38]. Biomass burning seems to contribute with the most part of the benzo[a]pyrene present in the environment [39]. The increase of this specific PAH in the pos-opening channel period may be associated with the higher input of terrestrial OM including some from biomass burning from agricultural areas of the Vale do Ribeira region. However, quantitative analysis of PAH is essential for evaluating the presence of this class in the environment studied.

## 4. Conclusions

In comparison with the traditional GC-MS technique, the alternative GC-HRTofMS one allows characterizing simultaneously a variety of geochemical biomarkers with reduction in both sample preparation time and number of chromatographic runs. The approach based on extraction with organic solvent and subsequent derivatization as well as in-situ derivatization thermal desorption can be used for such finality and deliver equivalent results. In particular, thermal desorption method requires few milligrams of sediment and no organic solvents. The geochemical biomarkers distributions along the sedimentary core collected at Valo Grande Channel revealed environmental changes occurred in the Cananéia-Iguape Estuarine-Lagunar System history.

## Data Availability

The data used to support the findings of this study are included within the article.

## Supplementary Materials

Supplementary Materials The Supplementary Material file contains the figure of the map showing the collection site at Cananéia-Iguape Estuarine-Lagunar System (São Paulo, Brazil).
