# Supplementary material for: Gas Chromatography Coupled to High Resolution Time-of-Flight Mass Spectrometry as a High-Throughput Tool for Characterizing Geochemical Biomarkers in Sediments
Source: Int J Anal Chem. 2018 Dec 2;2018:2560498. doi: 10.1155/2018/2560498 (PMC6304594; doi:10.1155/2018/2560498)
Supplement: Supplementary Materials — The Supplementary Material file contains the figure of the map showing the collection site at Cananéia-Iguape Estuarine-Lagunar System (São Paulo, Brazil). [file 2560498.f1.pdf]

Supplementary Material for:

Gas Chromatography Coupled to High Resolution Time-of-Flight Mass Spectrometry as a High-Throughput Tool for Characterizing Geochemical Biomarkers in Sediments

Hector Henrique Ferreira Koolen,[1,2*] Clécio Fernando Klitzke,[3] Joe Binkley,[3] Jeffrey Patrick,[3] Ana Cecília Rizatti de Albergaria-Barbosa,[4,5] Rolf Roland Weber,[5] Márcia Caruso Bícego,[5] Marcos Nogueira Eberlin,[1] Giovana Anceski Bataglion[1,6]

[1] ThoMSon Mass Spectrometry Laboratory, Institute of Chemistry, University of Campinas (UNICAMP), 13083-970, Campinas, SP, Brazil

[2] Metabolomics and Mass Spectrometry Research Group, Amazonas State University (UEA), 69065-001, Manaus, AM, Brazil

[3] LECO Corporation, 49085, St. Joseph, MI, USA

[4] Laboratory of Marine Geochemistry, Geoscience Institute, Federal University of Bahia (UFBA), 40170-020, Salvador, Bahia, Brazil

[5] Marine Organic Chemistry Laboratory, Oceanography Institute, University of São Paulo (USP), 05508-120, São Paulo, SP, Brazil

[6] Department of Chemistry, Federal University of Amazonas (UFAM), 69077-000, Manaus, AM, Brazil

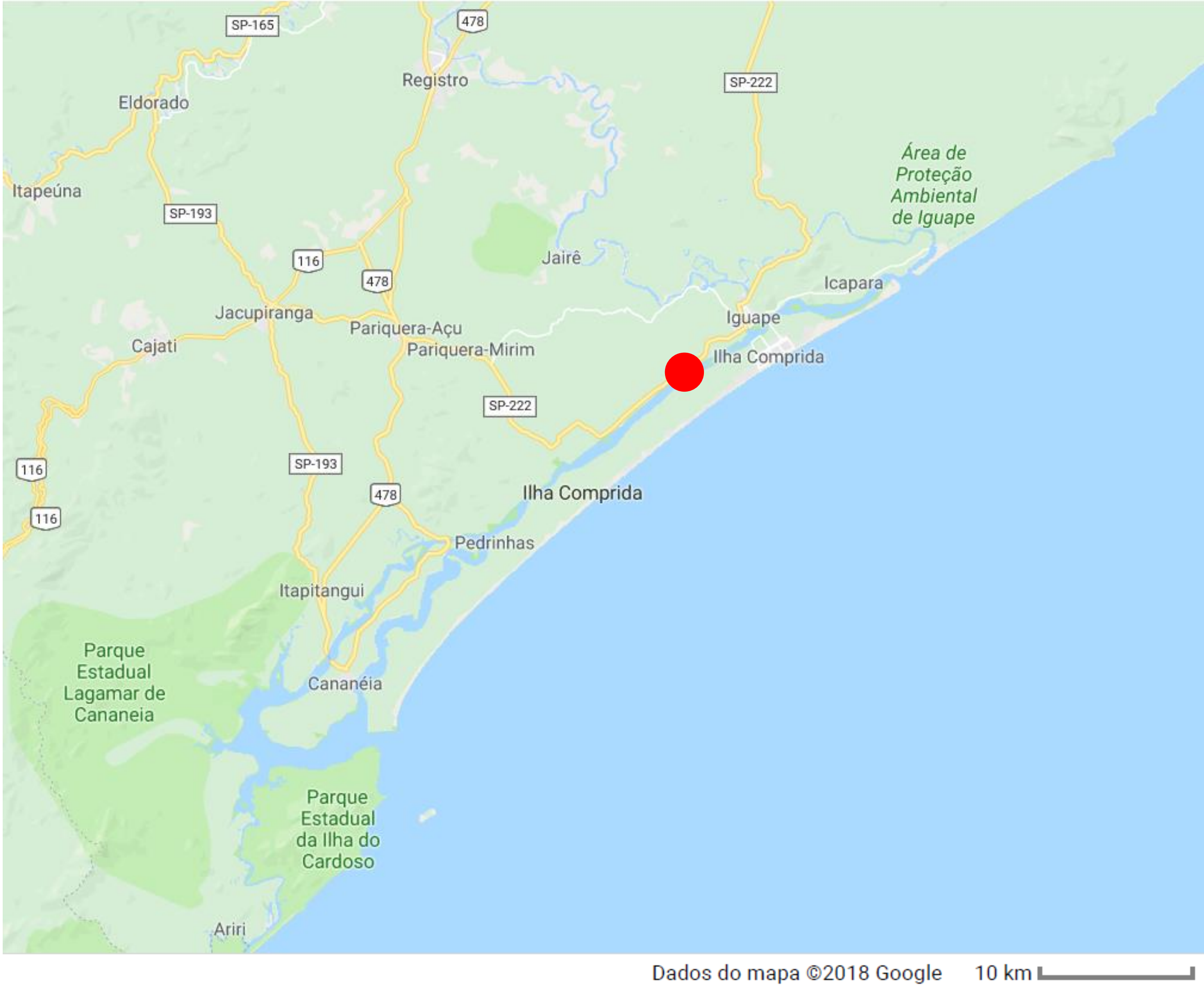

Figure S1. Study area (Cananéia-Iguape Estuarine-Lagunar System (São Paulo, Brazil)) and sampling station of sedimentary core in red, latitude and longitude of 24°45’2.45” S and 47°37’3.70” W, respectively.
